# Aborted Sudden Cardiac Death in a Female Patient Presenting with Takotsubo-Like Cardiomyopathy due to Epicardial Coronary Vasospasm

**DOI:** 10.1155/2017/7875240

**Published:** 2017-03-19

**Authors:** Sorin Giusca, Tom Eisele, Peter Nunninger, Benedikt Münz, Grigorios Korosoglou

**Affiliations:** ^1^Department of Cardiology & Vascular Medicine, GRN Hospital Weinheim, Weinheim, Germany; ^2^Department of Radiology, GRN Hospital Weinheim, Weinheim, Germany

## Abstract

Takotsubo cardiomyopathy is characterized by apical ballooning of the left ventricle (LV) in the absence of relevant coronary artery stenosis, which typically occurs in elderly women after emotional stress. Catecholamine cardiotoxicity, metabolic disturbance, and coronary microvascular impairment have previously been proposed as underlying pathophysiologic mechanisms of takotsubo cardiomyopathy, whereas myocardial stunning resulting from epicardial coronary artery vasospasm is not generally accepted as a cause of takotsubo cardiomyopathy. The prognosis of takotsubo cardiomyopathy is generally more favourable compared to myocardial infarction; however, severe complications such as rupture of the LV and life-threatening arrhythmias may occur. Herein, we describe a case of an 84-year-old female, who presented with aborted sudden cardiac death due to ventricular fibrillation. Echocardiography suggested LV apical ballooning with severely impaired LV-function, so that takotsubo cardiomyopathy was suspected. However, coronary angiography revealed epicardial spasm of the left anterior ascending, which resolved after intracoronary injection of 0.2 mg nitroglycerine. Cardiac magnetic resonance exhibited subendocardial late enhancement and echocardiography showed normalization of LV dysfunction during follow-up. The patient was put on conservative treatment with nitrates and calcium inhibitors and ICD implantation were deferred.

## 1. Introduction

Takotsubo cardiomyopathy, stress induced cardiomyopathy, or apical ballooning syndrome is a condition characterized by reversible myocardial dysfunction usually following a physical or emotional stress in the presence of nonobstructive epicardial arteries [1]. Myocardial dysfunction is typically located apically, although dysfunction of the mid-wall segments or basal segments have also been reported [2]. It mostly affects elderly women [3]. The clinical presentation has the hallmarks of an acute coronary syndrome, with patients describing acute chest pain, the ECG showing ischemic changes, blood tests revealing increased troponin levels, and cardiac imaging depicting regional wall motion abnormalities [4–6]. However, the clinical picture can vary greatly from mildly symptomatic patients to patients with cardiogenic shock and life-threatening ventricular arrhythmias [7].

Although several hypotheses were proposed for explaining the underlying pathophysiologic mechanisms, uncertainties remain regarding the pathogenesis of takotsubo cardiomyopathy. Increased sympathetic activation that leads to myocardial stunning, metabolic disturbances with increased oxidative stress at the level of the coronary endothelium, and disturbances of the coronary microcirculation were all described as possible pathomechanisms in this form of cardiomyopathy [8–10]. Coronary vasospasm was first thought to play a major role in the development of takotsubo cardiomyopathy [11]. However, the significance of vasospasm is currently rated differently in this context [12] and is rather not regarded as a central mechanism with takotsubo cardiomyopathy [7].

In most patients, the left ventricular (LV) function recovers spontaneously, although more recent observations revealed an increased risk of complications associated to takotsubo cardiomyopathy similar to that seen in patients with typical acute coronary syndrome [13].

We herein report the case of an 84-year-old female patient presenting with apical ballooning syndrome due to coronary vasospasm of the left anterior descending (LAD) coronary artery.

## 2. Case Presentation

**Figure 1:**
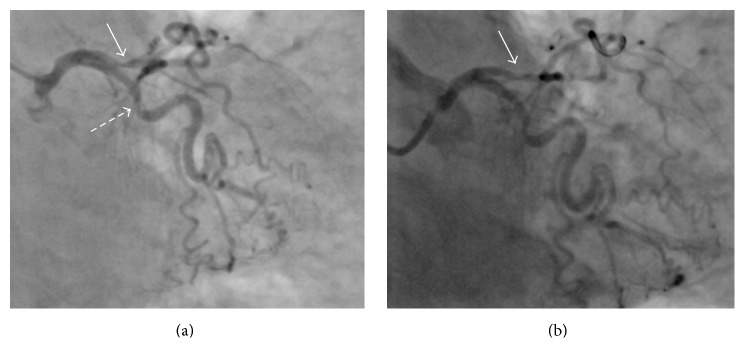
Coronary angiography, right anterior oblique view. (a) Note the presence of a relevant stenosis of the proximal segment of the left anterior descending artery (continuous arrow) and a moderate stenosis of the circumflex artery. (b) After the intracoronary injection of nitroglycerin not the reversibility of the left anterior descending stenosis.

An 84-year-old female patient was referred to our department after aborted sudden cardiac death due to ventricular fibrillation. The patient had experienced severe first time chest pain during the last hours prior to admission. Shortly before her arrival to our hospital, she developed ventricular fibrillation that was successfully converted to normal rhythm after application of a 200 J electrical shock by the emergency physician. On admission in our intensive care unit, she was awake, oriented, and hemodynamically stable. The patient's history revealed mild hypertension treated with a low-dose ACE-Inhibitor. No other cardiovascular risk factors were identified and no history of angina pectoris or exercise induced dyspnoea was reported. The ECG showed a sinus rhythm with T-wave inversion in the precordial leads (V1–V4). A bed-side echocardiography identified a severely reduced ejection fraction with an apical ballooning appearance of the LV. No signs of psychologic or physical stress were reported. The laboratory tests identified on admission elevated troponin levels (high sensitivity troponin = 102.7 ng/L) and increase white blood cells (13,000/*μ*l) with normal C-reactive protein. No other pathological changes in laboratory tests were found on admission. A coronary angiography was performed within the same day that revealed a moderate stenosis of the left circumflex coronary artery (dashed arrow in Figure 1(a)) as well as a localized spasm of the proximal LAD (white arrow in Figure 1(a)), which was reversible after intracoronary injection of 0.2 mg nitroglycerin (white arrow in Figure 1(b)).

**Figure 2:**
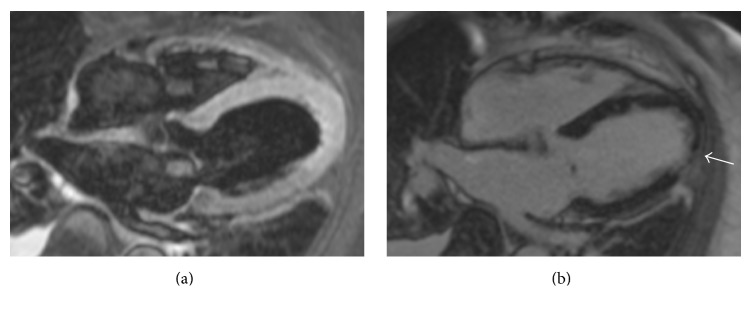
Cardiac MR performed two weeks after the coronary angiography showing (a) apical edema in a T2 weighted TIRM black blood acquisition and (b) subendocardial late gadolinium enhancement in the apical region.

The patient was treated with aspirin, statin, selective ß-blocker (bisoprolol), nitrate, and calcium antagonists. In addition, intravenous therapy with diuretics was initiated. Repeated echocardiographic examinations showed a slowly improving LV function with persistent hypokinesia of the apex. At one week, the LV ejection fraction was moderately reduced (ejection fraction of 42%). During the monitoring period, no ventricular arrhythmias were noted. Two weeks after the coronary angiography, a cardiac MRI was performed. The result of the examination was suggestive of the diagnosis of takotsubo cardiomyopathy with moderately reduced LV ejection fraction (Ejection fraction 44%), presence of myocardial edema in the apical region, and the presence of subendocardial late gadolinium enhancement in the apical region (Figures 2(a) and 2(b)). The patient recovered well and could be discharged after 21 days in stable condition and without clinical symptoms such as angina or dyspnea. Due to the episode of ventricular fibrillation and incomplete recovery of the LV function she was discharged with a life-vest. The subsequent Holter-ECG monitoring did not show any ventricular arrhythmia and the 1-month follow-up echocardiographic exam exhibited normal LV function (ejection fraction 61%) without wall motion abnormalities. Thus, the life-vest was removed and the ICD implantation could be deferred. The patient is in stable condition and without cardiac symptoms on three months of follow-up.

## 3. Discussion

Since its first description in 1990 in Japan [14], takotsubo cardiomyopathy has become a recognized clinical entity of acute heart failure syndrome. The main characteristic of this condition is the reversible myocardial dysfunction that arises in the presence of permeable coronary arteries. This form of cardiomyopathy is usually seen in elderly women as it was the case in our patient. Although an emotional or physical stress was thought to cause this syndrome, newer data suggest that in almost one-third of patients no trigger can be identified [13]. The clinical presentation can also vary from mild forms to cardiogenic shock and cardiac arrest. The latter was noted in our patient as ventricular fibrillation occurred during the transportation to the hospital. Moreover, our patient shares other common features of takotsubo cardiomyopathy. ECG changes, especially T-wave inversions, are a common feature of takotsubo cardiomyopathy, appearing in almost 40% of patients with this clinical entity [15]. Furthermore, similarly to our patient, the majority of patients diagnosed with takotsubo cardiomyopathy present with elevated levels of cardiac biomarkers on admission [16]. Moreover, the localization of the wall motion abnormalities is usually apical, although around 20% of patients can present mid-wall or basal patterns of ventricular dysfunction [2].

Interestingly, coronary angiography revealed a spasm of the LAD artery that was reversible immediately after the intracoronary injection of nitroglycerine. The pathophysiology of takotsubo cardiomyopathy is complex and not completely understood. A hyperactivity of the sympathetic system leading to increased catecolamine drive together with altered cellular metabolism and dysfunction of the coronary microcirculation is currently regarded as the main pathomechanisms, which lead to potentially reversible myocardial injury [7]. However, coronary vasospasm is a potential adjacent mechanism that could potentiate the previous described pathogenesis. Moreover, the first cases of takotsubo cardiomyopathy suggested coronary vasospasm as the underlying cause of apical myocardial dysfunction [11]. Furthermore, recent reports indicate that coronary vasospasm can act as a trigger for myocardial stunning compatible with the takotsubo syndrome [17, 18]. In our patient, we hypothesize that coronary vasospasm was the major trigger for developing reversible myocardial dysfunction of the LV-apex, compatible with takotsubo cardiomyopathy.

Alternatively, the takotsubo-like appearance of the LV could have been the result of the cardiac arrest which was triggered by coronary vasospasm causing subsequent myocardial ischemia. In addition, a prolonged vasospasm may have resulted in similar symptoms. However, some elements may not match with the hypothesis of prolonged coronary vasospasm as substrate for the clinical picture of the patient. Thus, the ECG changes were not typical for isolated vasospasm, which is usually associated with reversible ST-segment elevation [19]. In addition, with coronary vasospasm, myocardial dysfunction is usually reversible within seconds or minutes after the resolution of spasm, which was not the case in our patient [20]. Furthermore, our patient exhibited apical ballooning, which is typical for takotsubo cardiomyopathy and not with hypo- or akinesia of the anterior wall and of the apex, which would rather be typical for spasm of the LAD.

Cardiac MRI is a valuable tool in the diagnosis work-up of patients with takotsubo cardiomyopathy and can help differentiating between various clinical conditions. Although, at first, it was thought that the absence of late gadolinium enhancement was a condition for diagnosing takotsubo cardiomyopathy, more recent studies have identified patients diagnosed with this form of cardiomyopathy and exhibiting late gadolinium enhancement [21]. In addition, it appears that the late gadolinium enhancement is less likely to be identified in patients with takotsubo cardiomyopathy in subsequent cardiac MRI examination performed after several weeks from the first diagnosis [22].

The therapeutic approach is not standardized for this pathology and usually supportive therapy is indicated. Beta blockers seem to counterbalance the increased catecolamine drive, although a quarter of patients who develop takotsubo cardiomyopathy are already on beta blocker [23]. In our patient, due to the underlying mechanism identified, we decided for a combination of ß-blockers, ACE inhibitors calcium blockers, and nitrates. The patient showed a good clinical response with gradual improvement and then normalization of her LV function. This is in concordance with previous reports that found a complete recovery at the ventricular function at follow-up [23]. However, care should be taken in such patients, as the prognosis may not be so favourable in some cases [13].
